# Tixagevimab–cilgavimab for preventing breakthrough COVID-19 in dialysis patients: a prospective study

**DOI:** 10.1093/ckj/sfae309

**Published:** 2024-10-18

**Authors:** Sarinya Boongird, Thatsaphan Srithongkul, Sethanant Sethakarun, Jackrapong Bruminhent, Sasisopin Kiertiburanakul, Arkom Nongnuch, Chagriya Kitiyakara, Suchai Sritippayawan

**Affiliations:** D ivision of Nephrology, Department of Medicine, Faculty of Medicine Ramathibodi Hospital, Mahidol University, Bangkok, Thailand; Division of Nephrology, Department of Medicine, Faculty of Medicine Siriraj Hospital, Mahidol University, Bangkok, Thailand; Bhumirajanagarindra Kidney Institute, Bangkok, Thailand; Division of Infectious Diseases, Department of Medicine, Faculty of Medicine Ramathibodi Hospital, Mahidol University, Bangkok, Thailand; Division of Infectious Diseases, Department of Medicine, Faculty of Medicine Ramathibodi Hospital, Mahidol University, Bangkok, Thailand; D ivision of Nephrology, Department of Medicine, Faculty of Medicine Ramathibodi Hospital, Mahidol University, Bangkok, Thailand; D ivision of Nephrology, Department of Medicine, Faculty of Medicine Ramathibodi Hospital, Mahidol University, Bangkok, Thailand; Division of Nephrology, Department of Medicine, Faculty of Medicine Siriraj Hospital, Mahidol University, Bangkok, Thailand

**Keywords:** breakthrough COVID-19, dialysis, prophylaxis, safety, tixagevimab–cilgavimab

## Abstract

**Background:**

The effectiveness of tixagevimab–cilgavimab as pre-exposure prophylaxis (PrEP) against breakthrough coronavirus disease 2019 (COVID-19) in dialysis patients remains uncertain due to limited data.

**Methods:**

In this multicenter prospective study, we enrolled vaccinated dialysis patients and divided them into two groups: a tixagevimab–cilgavimab group (received a 150 mg/150 mg intramuscular dose of tixagevimab–cilgavimab) and a control group (age-matched patients not receiving tixagevimab–cilgavimab). The primary outcome was the breakthrough COVID-19 rate at 6 months, whereas secondary outcomes included COVID-19-related hospitalization, intensive care unit admission, endotracheal intubation and mortality. The safety of tixagevimab–cilgavimab was assessed.

**Results:**

Two hundred participants were enrolled, with equal numbers in each group (*n* = 100 each). Baseline characteristics were comparable between groups, except for a higher number of COVID-19 vaccine doses in the tixagevimab–cilgavimab group [median (IQR) 4 (3–5) vs. 3 (3–4); *P* = .01]. At 6 months, the breakthrough COVID-19 rates were comparable between the tixagevimab–cilgavimab (17%) and control (15%) groups (*P* = .66). However, the median (IQR) time to diagnosis of breakthrough infections tended to be longer in the tixagevimab–cilgavimab group [4.49 (2.81–4.98) vs 1.96 (1.65–2.91) months; *P* = .08]. Tixagevimab–cilgavimab significantly reduced COVID-19-related hospitalization rates (5.9% vs 40.0%; *P* = .02) among participants with breakthrough infections. All tixagevimab–cilgavimab-related adverse events were mild.

**Conclusion:**

The use of tixagevimab–cilgavimab as PrEP in vaccinated dialysis patients during the Omicron surge did not prevent breakthrough infections but significantly reduced COVID-19-related hospitalizations. Further research should prioritize alternative strategies.

KEY LEARNING POINTS **What was known:**

- Patients with end-stage kidney disease (ESKD) undergoing dialysis have an increased risk of severe acute respiratory syndrome coronavirus 2 (SARS-CoV-2) infection and severe complications due to compromised immunity and reduced immune response to vaccination.
- Tixagevimab–cilgavimab, a long-acting antibody (LAAB) combination, has been shown to effectively prevent symptomatic coronavirus disease 2019 (COVID-19) in unvaccinated high-risk individuals.
- However, the effectiveness and safety of tixagevimab–cilgavimab as pre-exposure prophylaxis (PrEP) in reducing breakthrough symptomatic COVID-19 and severe outcomes in vaccinated adult ESKD patients on dialysis remain uncertain.

**This study adds:**

- Tixagevimab–cilgavimab administration did not fully prevent breakthrough symptomatic COVID-19 during the Omicron variant's dominance.
- Its use as pre-exposure prophylaxis was associated with a significantly lower rate of COVID-19-related hospitalizations in patients who experienced breakthrough infections.
- The most common adverse events following administration were injection-site pain, followed by fatigue and fever.

**Potential impact:**

- Tixagevimab–cilgavimab as PrEP may be a valuable tool to mitigate severe COVID-19 outcomes in high-risk dialysis patients, even in the presence of breakthrough infections.
- The rapid emergence and mutation of Omicron sublineages during the study underscore the need for continuous monitoring of LAAB effectiveness, particularly next-generation LAABs, against evolving COVID-19 variants.

## INTRODUCTION

The coronavirus disease 2019 (COVID-19) pandemic has disproportionately affected immunocompromised individuals, including patients with end-stage kidney disease (ESKD) undergoing dialysis [1, 2]. Immunization demonstrates reduced efficacy in dialysis patients due to advanced age, comorbidities and inherent immunosuppression [3–7]. Despite prioritization for extended vaccination schedules and boosters, dialysis patients remain susceptible to breakthrough COVID-19 and severe outcomes, especially with emerging variants and waning immunity [3, 8]. Therefore, exploring alternative preventive strategies against COVID-19 is necessary for this vulnerable population.

Long-acting monoclonal antibodies (LAABs) have demonstrated efficacy as a pre-exposure prophylaxis (PrEP) against COVID-19, particularly in immunocompromised individuals with a blunted vaccine response [9, 10]. LAABs act by directly delivering antibodies that target the severe acute respiratory syndrome coronavirus 2 (SARS-CoV-2) spike protein, thereby blocking viral entry into cells [11, 12]. In the Phase 3 PROVENT (Safety and Efficacy of AZD7442, a Combination Product of Two Monoclonal Antibodies, for Pre-exposure Prophylaxis of COVID-19) trial, tixagevimab–cilgavimab, an established LAAB combination, demonstrated significant efficacy as a PrEP in preventing symptomatic COVID-19 in unvaccinated, high-risk adults over 6 months, with a relative risk reduction of 82.8% [95% confidence interval (CI) 65.8–91.4] [13]. However, a critical limitation of the PROVENT study was the underrepresentation of patients with chronic kidney disease (CKD); only 5% of the participants had CKD. Furthermore, there were no dialysis patients in this trial. These limitations restrict the generalizability of the findings of this trial to the broader CKD population, especially those on dialysis.

Previous studies have reported that tixagevimab–cilgavimab has varying efficacy in reducing breakthrough infections among patients with CKD, especially kidney transplant recipients (KTRs) and those receiving immunosuppressants [14–16]. However, specific data on dialysis patients remain scarce [17, 18]. This knowledge gap is concerning given the high risk of severe COVID-19 complications in dialysis patients [7]. Therefore, this study aimed to evaluate the effectiveness and safety of tixagevimab–cilgavimab as a PrEP in reducing breakthrough symptomatic COVID-19 and severe outcomes in vaccinated adult patients with ESKD on dialysis during the Omicron surge.

## MATERIALS AND METHODS

### Study design and participants

This multicenter prospective study enrolled adult patients with ESKD undergoing dialysis at three hospitals in Bangkok, Thailand: Ramathibodi Hospital (Mahidol University), Siriraj Hospital (Mahidol University) and Bhumirajanagarindra Kidney Institute Hospital. Enrollment occurred between November 2022 and February 2023, with follow-up extending to September 2023. This period coincided with the outbreak of Omicron lineages BA.2.75, XBB.1.5 and XBB.1.6 in Thailand.

The inclusion criteria were as follows: meeting standard dialysis adequacy requirements (weekly urea Kt/V ≥1.2 for hemodialysis and ≥1.7 for peritoneal dialysis), minimum weight of 40 kg and documented completion of an extended COVID-19 vaccination series (≥3 doses) at least 2 weeks prior as per Thai guidelines for tixagevimab–cilgavimab use during the study period. Individuals with documented contraindications to vaccination or incomplete vaccination schedules were considered for inclusion based on physician's assessment of the potential benefit–risk ratio. The exclusion criteria were as follows: confirmed COVID-19 in the past 3 months, pregnancy or breastfeeding, active respiratory tract infection, life expectancy below 6 months, and recent diagnosis (within 6 months) of severe heart disease or uncontrolled arrhythmias.

Before enrollment, all potential participants underwent a screening process including questionnaires to assess current respiratory tract symptoms and potential COVID-19 exposure, followed by a rapid antigen test for SARS-CoV-2 infection using a European Union–approved test with a reported sensitivity of 97.2% and a specificity of 99.0% [19].

We used Stata 18 to calculate the sample size, assuming a two-sided alpha of 0.05 and power of 80%. Based on Thai dialysis patient data in 2021 [20] and the PROVENT study [13], we anticipated COVID-19 rates of 15% and 3% in the placebo and tixagevimab–cilgavimab groups at 6 months, respectively, which necessitated the enrollment of 89 patients in each group. To account for a potential 10% dropout rate, we enrolled a total of 200 participants (100 participants per group).

### Intervention and follow-up

Following the eligibility assessment, the participants were informed about tixagevimab–cilgavimab (Evusheld^®^, AstraZeneca). The participants were offered a choice between receiving tixagevimab–cilgavimab and participating in the control group (patient-directed assignment). The participants in the tixagevimab–cilgavimab group received two intramuscular injections of 150 mg/150 mg tixagevimab–cilgavimab (1.5 mL each) at the participating centers. Those who declined receiving tixagevimab–cilgavimab were age-matched (1:1) with participants in the tixagevimab–cilgavimab group based on the enrollment date (control group). Follow-up for controls began on the same day as their matched counterparts in the tixagevimab–cilgavimab group.

Baseline data at enrollment included demographics, vaccination status, laboratory results and quantitative antireceptor binding domain of SARS-CoV-2 immunoglobulin G antibody (anti-RBD IgG) levels. Anti-RBD IgG levels were measured using the Abbott SARS-CoV-2 IgG II Quantification assay (Abbott Diagnostics, Lake Bluff, IL, USA) on the Abbott Alinity system following the manufacturer's instructions.

Following tixagevimab–cilgavimab administration, the participants were observed for 1 h at the dialysis center to monitor for immediate adverse events (AEs). This was followed by scheduled phone calls on Days 3 and 7 to assess AEs. All participants in both groups were prospectively followed up for 6 months, with monthly contact to assess for SARS-CoV-2 infection, severe clinical outcomes, and any AEs. The participants were instructed to use self-administered rapid antigen test kits if they experienced any suspected COVID-19 symptoms, regardless of severity, had been exposed to confirmed COVID-19 cases or had any concerns.

### Outcomes

#### Efficacy outcomes

The primary efficacy outcome was the cumulative incidence of breakthrough symptomatic COVID-19, as confirmed through reverse transcription polymerase chain reaction testing for SARS-CoV-2, in the tixagevimab–cilgavimab group compared with that in the control group during the 6-month follow-up period.

To assess the effectiveness of tixagevimab–cilgavimab in preventing severe COVID-19 outcomes, we evaluated two secondary efficacy endpoints. First, time-to-event analysis investigated the duration until the first occurrence of breakthrough COVID-19. Second, we evaluated the incidence rates and event-free probabilities of severe COVID-19 outcomes—specifically COVID-19-related hospitalization, intensive care unit (ICU) admission, endotracheal intubation and 28-day mortality during follow-up. Independent physicians who were blinded to the study determined the management of breakthrough infections based on local treatment protocols and individual patient assessments. Only the first breakthrough COVID-19 per participant was included in the analysis.

#### Safety outcomes

Safety was assessed by monitoring the incidence of all reported and medically attended AEs, including systemic and local AEs, in the tixagevimab–cilgavimab group. Data collection was performed on the day of tixagevimab–cilgavimab administration (Day 0), Day 3 and Day 7.

### Ethical considerations

The study protocol and other relevant documentation received ethical approval from the Institutional Review Board (IRB) at each participating site (approval number: MURA 2022/513 for Ramathibodi Hospital; Si 802/2022 for Siriraj Hospital; and IRB Ref. No. 3/2565 for Bhumirajanagarindra Kidney Institute Hospital). This study adhered to the ethical principles established in the Declaration of Helsinki. Written informed consent was obtained from all participants before enrollment. This study was registered with the Thai Clinical Trials Registry (No. TCTR20221031004).

### Statistical analysis

Baseline characteristics are presented according to the treatment group. Categorical variables are presented as frequencies and percentages, and continuous variables are presented as means with standard deviations (SD) if normally distributed or medians with interquartile ranges (IQR) if not normally distributed. Categorical variables were compared using chi-square tests, with Fisher's exact test applied for small samples. Continuous variables were analyzed using Student's *t*-test for normally distributed data and Mann–Whitney U test for non-normally distributed data.

Kaplan–Meier curves were plotted for the cumulative incidence of breakthrough symptomatic COVID-19 and the event-free probabilities for COVID-19-related hospitalization, ICU admission, endotracheal intubation and death. Log-rank tests were used for group comparisons. The frequency of AEs associated with tixagevimab–cilgavimab administration is summarized using descriptive statistics. Statistical significance was set at a *P-*value of <.05. Data analysis and visualization were performed using Stata 18.0 (Stata Corp., College Station, TX, USA).

## RESULTS

### Patient characteristics

**Figure 1:**
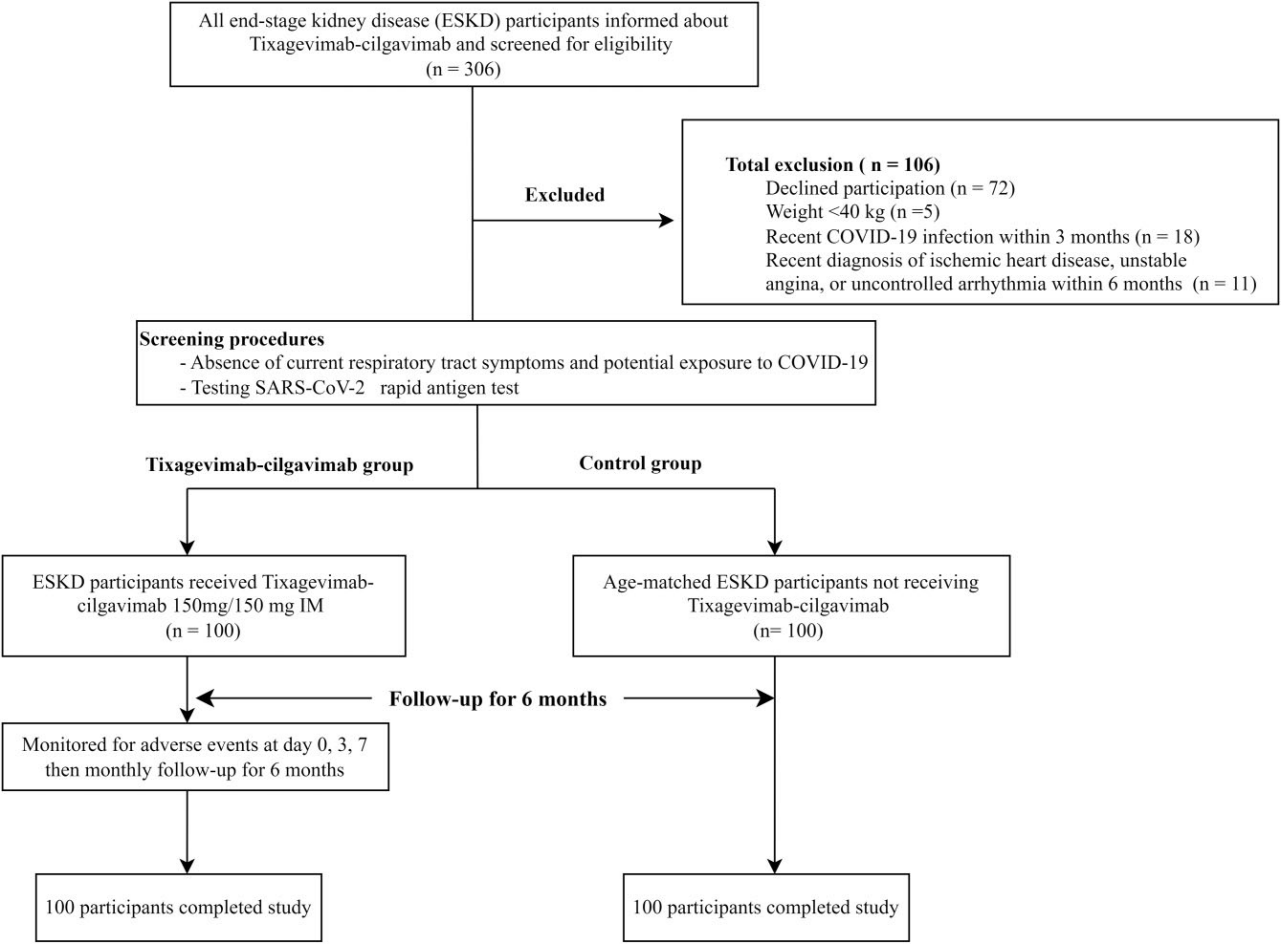
Study flowchart.

Of the 306 screened patients with ESKD on dialysis, 200 (100 per group) completed the study (Fig. 1), with a median follow-up duration of 6.32 months.

Baseline characteristics (Table 1) revealed similar demographics and clinical characteristics between groups, with the exception that the control group had a higher proportion of males (54% vs 39%, *P* = .03). Both groups had comparable mean ages (approximately 66 years), with more than 80% receiving hemodialysis. Prior COVID-19 was reported in 30% of participants in both groups.

**Table 1:** Baseline characteristics of participants.

Baseline characteristics	Total (*n* = 200)	Tixagevimab–cilgavimab group (*n* = 100)	Control group (*n* = 100)	*P*-value
Age, years, mean (SD)	66.0 (15.6)	66.2 (14.9)	65.8 (16.3)	.94
Male sex, *n* (%)	93 (46.5)	39 (39.0)	54 (54.0)	.03^a^
Weight, kg, mean (SD)	61.1 (14.3)	62.3 (16.6)	60.0 (11.6)	.26
Body-mass index, kg/m^2^, mean (SD)	23.0 (4.3)	23.3 (4.8)	22.7 (3.7)	.97
Dialysis modality, *n* (%)				
Hemodialysis	169 (84.5)	82 (82.0)	87 (87.0)	.33
Peritoneal dialysis	31 (15.5)	18 (18.0)	13 (13.0)	
Anuria, *n* (%)	71 (35.5)	40 (40.0)	31 (31.0)	.18
Cause(s) of ESKD, *n* (%)				.92
Diabetic nephropathy	83 (41.5)	40 (40.0)	43 (43.0)	
Hypertensive nephropathy	46 (23.0)	23 (23.0)	23 (23.0)	
Glomerulonephritis	23 (11.5)	13 (13.0)	10 (10.0)	
Others	48 (24.0)	24 (24.0)	24 (24.0)	
Comorbidities, *n* (%)				
Hypertension	193 (96.5)	98 (98.0)	95 (95.0)	.25
Dyslipidemia	140 (70.0)	70 (70.0)	70 (70.0)	1.00
Diabetes mellitus	101 (50.5)	51 (51.0)	50 (50.0)	.89
Coronary artery disease	41 (20.5)	17 (17.0)	24 (24.0)	.22
Cerebrovascular disease	18 (9.0)	8 (8.0)	10 (10.0)	.62
Congestive heart failure	15 (7.5)	6 (6.0)	9 (9.0)	.42
Chronic obstructive pulmonary disease	2 (1.0)	1 (1.0)	1 (1.0)	.75
Cancer	22 (11.0)	7 (7.0)	15 (15.0)	.07
Age-adjusted Charlson comorbidity index, mean (SD)	5.7 (2.1)	5.5 (1.9)	5.9 (2.3)	.24
Previous COVID-19, *n* (%)	62 (31.0)	31 (31.0)	31 (31.0)	1.00
Completed extended primary COVID-19 vaccination (≥3 doses), *n* (%)	177 (88.5)	94 (94.0)	83 (83.0)	.02^a^
Doses of COVID-19 vaccine received, median (IQR)	4 (3–5)	4 (3–5)	3 (3–4)	.01^a^
mRNA COVID-19 vaccine, *n* (%)	183 (91.5)	92 (92.0)	91 (91.0)	.80
Laboratories				
Hemoglobin, g/dL, mean (SD)	10.7 (1.3)	10.8 (1.1)	10.6 (1.4)	.22
White blood cell count, ×10^9^/L, mean (SD)	6.4 (2.2)	6.6 (2.1)	6.1 (2.3)	.04^a^
Percentage of neutrophil, %, mean (SD)	64.9 (8.9)	65.1 (9.4)	64.9 (8.7)	.82
Percentage of lymphocyte, %, mean (SD)	22.2 (7.3)	21.5 (7.3)	22.9 (7.3)	.11
Blood urea nitrogen, mg/dL, mean (SD)	59.4 (19.6)	60.8 (17.9)	57.7 (21.1)	.31
Sodium, mmol/L, mean (SD)	137.2 (3.2)	137.6 (2.8)	136.9 (3.5)	.16
Potassium, mmol/L, mean (SD)	4.4 (0.7)	4.6 (0.6)	4.3 (0.7)	.01^a^
Albumin, g/L, mean (SD)	38.0 (5.3)	39.2 (5.2)	36.8 (5.2)	<.01^a^
Intact parathyroid hormone, pg/mL, median (IQR)	339.0 (166.0–523.0)	347.6 (178.7–658.4)	311.0 (165.0–463.0)	.30
Serum ferritin, ng/mL, median (IQR)	474.0 (234.0–749.3)	398.3 (212.8–697.0)	545.5 (265.5–823.5)	.09
Anti-SARS-CoV-2 spike RBD IgG Ab levels, BAU/mL, median (IQR)	5827.5 (2103.8–18086.1)	6294.2 (2833.4–19413.6)	5722.7 (1693.4–17021.8)	.30

Data are presented as mean and standard deviation (SD) unless otherwise specified. Baseline characteristics were compared between groups. Continuous variables were assessed using Student's *t*-test for normally distributed data and the Mann–Whitney U test for non-normally distributed data. Categorical variables were compared using Fisher's exact test.

a *P-*value <.05.

The body mass index was calculated from weight in kilograms divided by height squared; anuria was defined as passing urine output below 100 mL per day; total Kt/VUrea represented total small-solute urea clearances.

BAU, binding antibody units.

The tixagevimab–cilgavimab group received significantly more COVID-19 vaccine doses [median (IQR) 4 (3–5) vs. 3 (3–4); *P* = .01] and had a higher completion rate for the extended primary series (≥3 doses) (94% vs 83%, *P* = .02) than the control group.

The baseline laboratory values were similar between the groups. However, the tixagevimab–cilgavimab group exhibited higher total white blood cell counts and serum potassium and albumin levels than the control group. The baseline anti-RBD IgG levels were comparable between groups.

### Breakthrough symptomatic COVID-19

**Figure 2:**
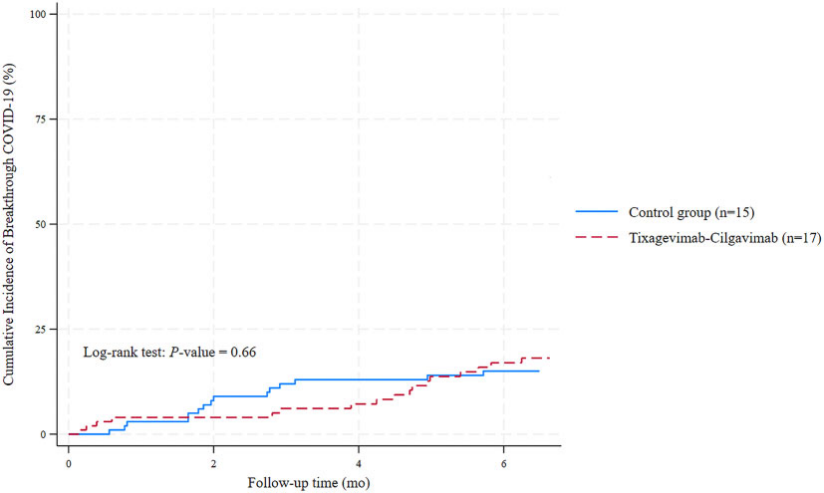
Cumulative incidence of breakthrough COVID-19 in the tixagevimab–cilgavimab and control groups over 6-month follow-up.

At 6 months, breakthrough symptomatic COVID-19 occurred in 32 participants (16%), with comparable cumulative incidence rates in the tixagevimab–cilgavimab and control groups (17% vs 15%, *P* = 0.66) (Fig. 2). Although the initial breakthrough rates at 3 months appeared lower in the tixagevimab–cilgavimab group than in the control group (6% vs 13%), they converged by six months (17% vs 15%). Consistent with this observation, the incidence rate ratio (IRR) revealed no protective effect of tixagevimab–cilgavimab (IRR 1.17; 95% CI 0.55–2.51; *P* = .79) (Table 2).

**Table 2:** Incidence rates and comparisons of breakthrough symptomatic COVID-19 and severe COVID-19 outcomes in the tixagevimab–cilgavimab and controls.

	Total (*n* = 200)			Tixagevimab–cilgavimab group (*n* = 100)			Control group (*n* = 100)				
Event	No./total no. (%)	Total person-months	Incidence rate (95% CI)	No./total no. (%)	Total person-months	Incidence rate (95% CI)	No./total no. (%)	Total person- months	Incidence rate (95% CI)	IRR (95% CI)	*P*-value
Primary efficacy outcome											
Breakthrough symptomatic COVID-19	32/200 (16.0)	1121.7	0.029 (0.020–0.040)	17/100 (17)	552.3	0.031 (0.019–0.050)	15/100 (15)	569.5	0.026 (0.016–0.044)	1.169 (0.549–2.513)	.79
Secondary efficacy outcomes											
Hospitalization among breakthrough symptomatic COVID-19 cases	7/32 (21.9)	97.5	0.072 (0.034–0.151)	1/17 (5.9)	62.2	0.016 (0.002–0.114)	6/15(40.0)	35.3	0.170 (0.076–0.379)	0.094 (0.002–0.779)	.02^a^
ICU admission among breakthrough symptomatic COVID-19 cases	2/32 (6.3)	97.5	0.021 (0.005–0.082)	1/17 (5.9)	62.2	0.016 (0.002–0.114)	1/15 (6.7)	35.3	0.028 (0.004–0.201)	0.567 (0.007–44.495)	.99
Endotracheal intubation among breakthrough symptomatic COVID-19 cases	1/32 (3.1)	97.5	0.010 (0.001–0.073)	0 (0)	62.2	0	1/15 (6.7)	35.3	0.028 (0.004–0.201)	0 (0–22.107)	.72
COVID-19-related death among breakthrough symptomatic COVID-19 cases	1/32 (3.1)	97.5	0.010 (0.001–0.073)	0 (0)	62.2	0	1/15 (6.7)	35.3	0.028 (0.004–0.201)	0 (0– 22.107)	.72

The IRR was calculated by dividing the incidence rate of the tixagevimab-cilgavimab group by the incidence rate of the control group.

a *P*-value <.05.

Among participants who developed breakthrough infection, the median (IQR) time to breakthrough COVID-19 tended to be longer in the tixagevimab–cilgavimab group [4.49 (2.81–4.98) months] than in the control group [1.96 (1.65–2.91) months] (*P* = .08).

### Severe COVID-19-related outcomes

**Figure 3:**
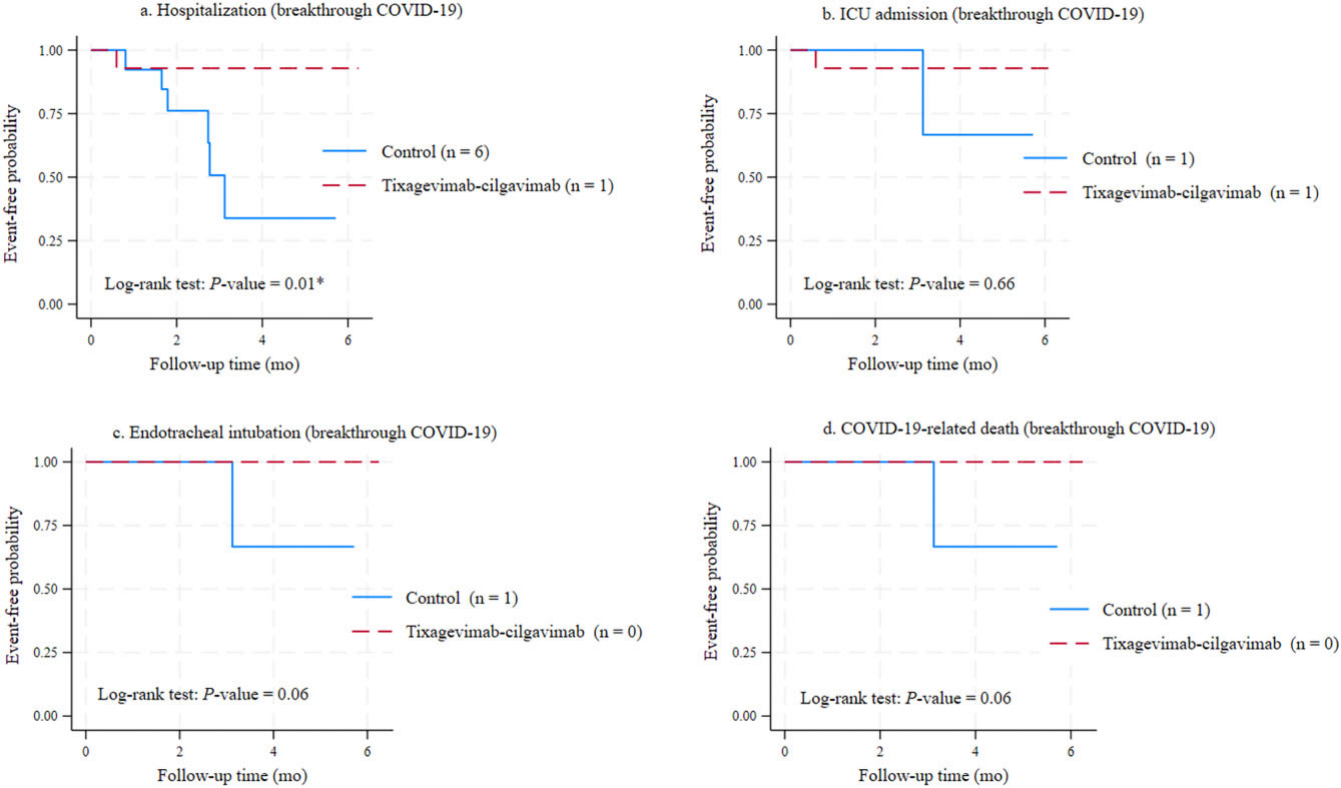
Event-free probabilities of severe COVID-19 outcomes among breakthrough cases. (a) Hospitalization. (b) ICU admission. (c) Endotracheal intubation. (d) COVID-19-related death.

**Figure 4:**
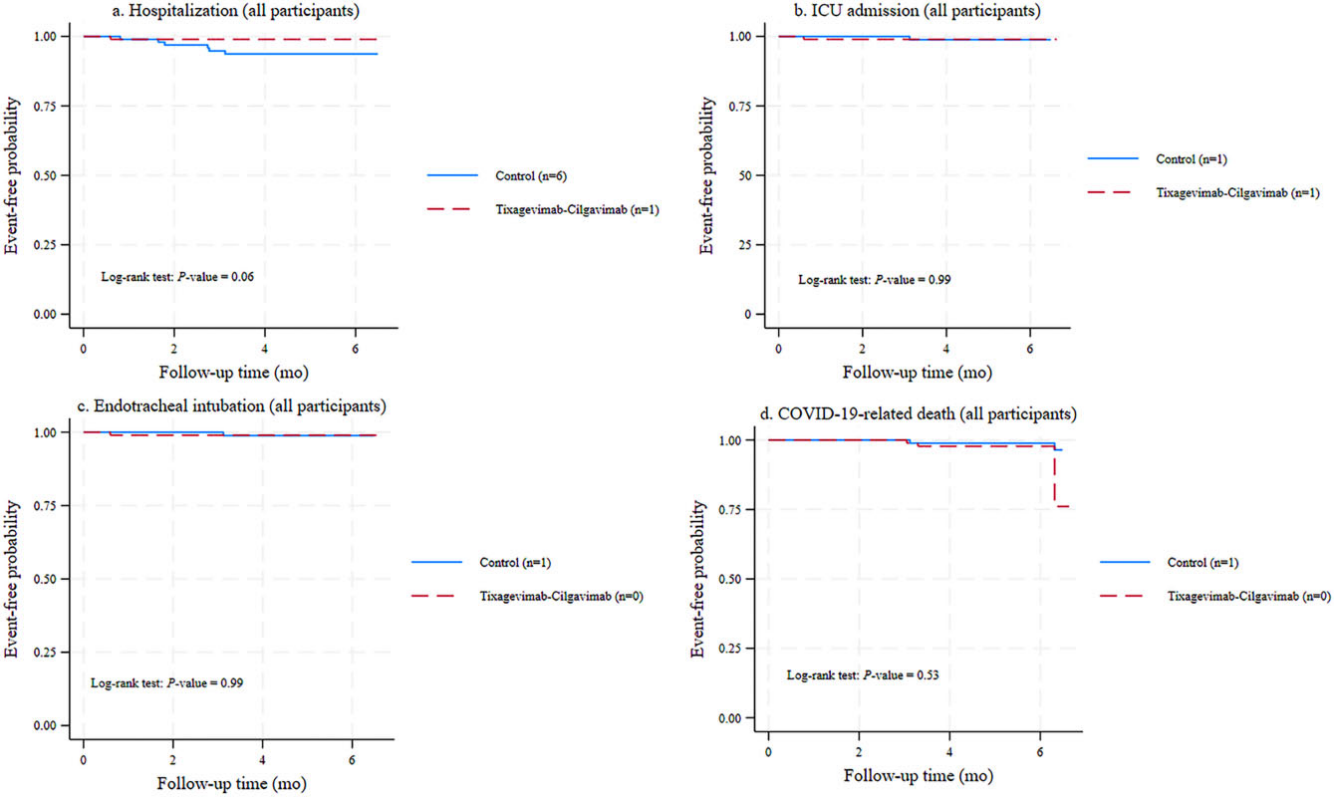
Event-free probabilities of severe COVID-19 outcomes in the entire study population. (a) Hospitalization. (b) ICU admission. (c) Endotracheal intubation. (d) COVID-19-related death.

Figures 3a–d and 4a–d depict the event-free probabilities for hospitalization, ICU admission, endotracheal intubation and death from COVID-19. Figure 3 focuses on participants who experienced breakthrough infection, whereas Fig. 4 presents data for the entire study population during the 6-month follow-up.

#### Breakthrough cases

Breakthrough COVID-19 resulted in hospitalization for 7 of 32 participants (21.9%). The tixagevimab–cilgavimab group experienced a significantly lower hospitalization rate (5.9%) than the control group (40.0%) (*P* = .01) (Table 2 and Fig. 3a), which translates to a 91% reduction in hospitalization risk (IRR 0.094; 95% CI 0.002–0.779; *P* = .02).

Two hospitalized participants, one from each group, required ICU admission (Fig. 3b). No significant difference in event-free probabilities for ICU admission was observed (*P *= .66).

One participant from the control group required ICU admission and endotracheal intubation and subsequently died of COVID-19. No deaths or endotracheal intubation events occurred in the tixagevimab–cilgavimab group. Although not statistically significant (*P *= .06), Kaplan–Meier curves (Fig. 3c and d) suggested trends toward lower probabilities of severe outcomes in the tixagevimab–cilgavimab group.

#### All participants

The tixagevimab–cilgavimab group exhibited a trend toward lower COVID-19-related hospitalization risk compared with the control group (*P *= .06) (Fig. 4a). However, the event-free probabilities for COVID-19-related ICU admission, endotracheal intubation and death did not differ between groups (Fig. 4b–d).

### Safety and AEs

At the end of the follow-up period, 37 participants (37%) in the tixagevimab–cilgavimab group reported at least one AE (Table 3). Injection-site pain (16%), fatigue (15%) and fever (6%) were the most frequent AEs. All reported AEs were mild, resolved within 7 days, and did not necessitate medical attention.

**Table 3:** AEs following tixagevimab–cilgavimab administration.

	Day 0	Day 3	Day 7
Event	(*n* = 100)	(*n* = 100)	(*n* = 100)
Medically attended AEs	0	0	0
Pain at the injection site	16	3	0
Fatigue	15	4	0
Fever	6	0	0
Headache	5	0	0
Nausea	1	0	0

One participant can experience multiple events.

## DISCUSSION

In this prospective, multicenter study, we evaluated the effectiveness and safety of tixagevimab–cilgavimab as a PrEP in preventing breakthrough symptomatic COVID-19 in adult patients with ESKD on dialysis during the Omicron surge. Although tixagevimab–cilgavimab did not reduce the overall number of breakthrough infections at 6 months, it significantly reduced the hospitalization rate among participants who experienced breakthrough COVID-19. Moreover, the results suggested potential benefits of tixagevimab–cilgavimab in reducing ICU admissions, endotracheal intubation and death. All reported early AEs associated with tixagevimab–cilgavimab were mild.

Earlier studies have reported encouraging results for tixagevimab–cilgavimab in preventing breakthrough COVID-19 in high-risk populations [13, 16, 21]. These studies were primarily conducted during the outbreak of variants against which tixagevimab–cilgavimab retains neutralizing activity, such as Alpha or BA.1. However, we observed no significant reduction in breakthrough rates in vaccinated patients on dialysis who received tixagevimab–cilgavimab (300 mg) compared with controls. This finding suggests the influence of rapidly evolving viral variants. Notably, our study period coincided with a shift from BA.2.75 to XBB.1.5 and XBB.1.6 as the dominant variants, which are known for their immune escape capabilities [22]. An initial 7% reduction in breakthrough rates within the tixagevimab–cilgavimab group suggested some susceptibility of earlier variants, such as BA.2.75 and BA.5, to tixagevimab–cilgavimab. However, these benefits diminished with the emergence of XBB.1.5 and XBB.1.6, against which tixagevimab–cilgavimab may have reduced or lost efficacy, as supported by *in vitro* data [23, 24]. The limited previous data on the use of tixagevimab–cilgavimab in dialysis patients reveal conflicting results [17, 18]. Khan *et al*. [17] reported substantially lower breakthrough infections (7% vs 57%) and hospitalization rates (2% vs 43%) with a higher tixagevimab–cilgavimab dose (600 mg) compared with those in controls. Conversely, Nassar *et al*. [18] observed similar overall breakthrough rates in hemodialysis patients receiving a lower dose (300 mg) and controls (23% vs 20%; *P* = .59). These variations likely stem from differences in the study design, patient characteristics, dosage regimens and circulating viral variants [17, 18]. Supporting these observations, the breakthrough infection rate in our dialysis patients mirrored the national data for Thai dialysis patients during the same period, dominated by BA.2.75 and XBB.1.5/XBB.1.6 Omicron sublineages [25, 26]. This suggests that the 300 mg tixagevimab–cilgavimab dose may be less effective against these particular variants. The rapid mutation of Omicron sublineages during our study highlights the need for further research on the effectiveness of new monoclonal antibodies against emerging variants, particularly for high-risk populations such as dialysis patients.

Breakthrough infections remain a concern, and interventions that can mitigate severe COVID-19 outcomes even without complete infection prevention require further investigation. Our findings, along with previous studies [17, 18], suggest that tixagevimab–cilgavimab reduces severe outcomes in dialysis patients. Despite a limited number of severe events (*n* = 7), we observed a significant 10.6-fold decrease in hospitalization rates in the tixagevimab–cilgavimab group, with trends toward lower rates of endotracheal intubation and death among those who experienced breakthrough infections. These findings are consistent with previous studies demonstrating reduced hospitalization, ICU admission and death in KTRs and dialysis patients receiving tixagevimab–cilgavimab [14, 16–18, 21]. This observed benefit in the tixagevimab–cilgavimab group is likely attributable to the provision of passive immunization. Although *in vitro* neutralization against emerging variants may be reduced [27–29], the unique Fc region modification of tixagevimab–cilgavimab extends its presence in the bloodstream for up to 9 months [30]. This prolonged presence allows for immediate viral neutralization upon breakthrough infection [11, 31], potentially mitigating severe outcomes even when complete viral clearance is not achieved. While baseline anti-RBD IgG levels were comparable between groups, the higher vaccination dose in the tixagevimab–cilgavimab group remains a potential confounding factor. This may have contributed to a broader immune response not fully captured by the measured anti-RBD IgG levels. Consequently, the potential benefits of reduced COVID-19-related hospitalization observed in patients who received tixagevimab–cilgavimab in this and previous studies [17, 18] should be interpreted with caution. Ongoing trials of next-generation LAABs, such as AZD3152 (NCT05648110) [32, 33], hold promise for improved protection against breakthrough infections and severe outcomes in immunocompromised populations.

Evaluation of AEs following tixagevimab–cilgavimab administration in our dialysis cohort mirrored findings in other immunocompromised populations [21, 34] and prior dialysis studies [17, 18], with AEs being predominantly mild and transient. Injection-site pain was the most common AE reported across studies, followed by fatigue and fever [21, 34]. No anaphylaxis or tixagevimab–cilgavimab-related cardiovascular events occurred within 1 h or during follow-up, possibly due to our exclusion criteria, which restricted the participation of patients with uncontrolled cardiac conditions. However, a definitive assessment of the long-term safety profile of tixagevimab–cilgavimab in dialysis patients is needed.

The present study has several strengths. The inclusion of a concurrently enrolled, age-matched control group strengthened the study by minimizing confounding factors, such as age and temporal trends. Additionally, assessing baseline anti-RBD IgG levels in all participants allowed us to address confounding variables related to pre-existing humoral immunity. These baseline findings can inform future investigations aimed at developing more effective patient selection criteria. However, our study had several limitations. The relatively small sample size limited our ability to detect critical outcomes, such as hospitalization or death. The lack of randomization may have introduced potential imbalances in other aspects of pre-existing immunity beyond anti-RBD IgG, or unknown confounders, potentially affecting the results. Moreover, we did not assess cellular immunity against Omicron, which may be less impacted by viral mutations than humoral immunity and could influence outcomes [35]. Focusing solely on symptomatic infections may have overestimated tixagevimab–cilgavimab's overall effectiveness, particularly in asymptomatic or mildly symptomatic cases. Future studies should incorporate regular testing to capture infections more comprehensively, include assessments of both humoral and cellular immunity, and evaluate the cost-effectiveness of tixagevimab–cilgavimab as a preventive strategy. Additionally, using randomized designs and stratifying participants based on baseline anti-RBD IgG levels could help reduce potential bias and identify subgroups most likely to benefit from tixagevimab–cilgavimab prophylaxis.

In this prospective, multicenter trial, we investigated the effectiveness and safety of a 300-mg dose of tixagevimab–cilgavimab as PrEP against breakthrough symptomatic COVID-19 and severe outcomes in vaccinated dialysis patients during the Omicron variant surge. Although breakthrough infections occurred in both groups, tixagevimab–cilgavimab significantly reduced COVID-19-related hospitalization rates. However, the higher baseline vaccination in the tixagevimab–cilgavimab group warrants cautious interpretation of these observed benefits. Despite this, our findings suggest a potential effect of tixagevimab–cilgavimab in mitigating severe COVID-19 outcomes in this vulnerable population. Future research should focus on developing next-generation LAAB therapies with broader and more potent antiviral activity, which could improve COVID-19 prevention and outcomes in dialysis patients and potentially other immunocompromised populations.

## Data Availability

The datasets collected and/or analyzed during this study are not publicly available; however, anonymized data are available upon reasonable request from the corresponding author.
